# Lipid droplets and major metabolic disorders

**DOI:** 10.1007/s11033-026-11563-x

**Published:** 2026-02-13

**Authors:** William J. Dechert, Guanglong He

**Affiliations:** School of Pharmacy, College of Health Sciences, University of Wyoming, 1000 E. University Avenue, Laramie, WY 82071 USA

**Keywords:** Lipid droplets (LDs), Lipolysis, Lipophagy, Metabolism, Chronic inflammation

## Abstract

Lipid droplets (LDs) are ubiquitous subcellular organelles playing crucial roles in lipid and energy homeostasis. They are constantly generated in the intramembrane space of the endoplasmic reticulum (ER) through unique mechanisms. Upon maturation, they bud off from the ER outer membrane into the cytosol, travel through the cytosolic microtubular network, and make contacts to most of the other subcellular organelles to perform their cellular functions. On the one hand, these organelles can grow or fuse with other smaller ones and serve as storage for extra cellular lipid products. On the other hand, when metabolic needs arise such as in nutrient deprivation or during exercises, the esterified lipids inside the LDs undergo stepwise lipolysis, e.g. basal and stimulated lipolysis, under the regulation of a set of proteins and kinases that are specifically targeted to the monolayer phospholipid membrane of the LDs. The dynamic homeostasis of their biogenesis and lipolysis is also intimately related to other cellular signaling pathways in a paracrine or endocrine manner which actively participate in the regulation of cellular homeostasis and systemic health. This review will summarize the current understanding of the underlying mechanisms mediating the biogenesis and metabolic impact of LDs in normal and disease status with a focus on their roles in propelling and sustaining chronic inflammation.

## Introduction

Lipid droplets (LDs) play a critical role in lipid and energy homeostasis. These subcellular organelles possess unique mechanisms governing their biogenesis, growth and fusion, and lipolysis. LDs function both as scavengers of toxic lipids and sources of inflammation in various cells and tissues. LDs are derived from the endoplasmic reticulum (ER) with a set of ER-specific residential proteins in the synthesis of triglyceride (TAG) and sterol esters (SE) leading to lens-like structure formation through demixing [1]. Their monolayer phospholipid membranes are embedded with unique LD-specific proteins regulating their biogenesis, growth and fusion, lipolysis, transport, and interaction with other subcellular organelles [2]. When nutrients are in surplus, as in obesity, LDs accumulate abundantly not only in adipocytes, but also in almost every other type of cells. For example, in adipose tissue macrophages, the accumulated LDs demonstrate a dysregulated lipolysis which sustains the inflammatory signaling and drives continuous inflammatory responses with detrimental consequences on cardiovascular, hepatic, and neuronal systems [3]. However, the mechanisms underlying these chronic inflammatory responses are still not completely understood. In neurodegenerative disorders such as Alzheimer’s disease (AD), Parkinson’s disease (PD), Huntington’s disease (HD), amyotrophic lateral sclerosis (ALS), and multiple sclerosis (MS), LDs accumulate in microglia and other neurites with disease progression within the brain, which is rich in lipids [4–6]. However, our understanding of the role of the accumulated LDs in the activated microglia and related inflammation underlying these neurodegenerative disorders remains incomplete. The regulatory roles of LDs and their impact on other cellular systems such as cardiomyocytes, skeletal muscle cells, endothelial cells, and hepatocytes have just been recognized. Our understanding of this unique organelle has advanced beyond its conventional role as a lipid storage unit to its mechanistic functions in health and diseases. This review will provide an overall summary of our current understanding of LD biology and potential implications in therapeutic interventions for treating related lipid metabolism disorders due to dysregulated lipolysis and inflammation.

## Lipid droplet biology

**Fig. 1:**
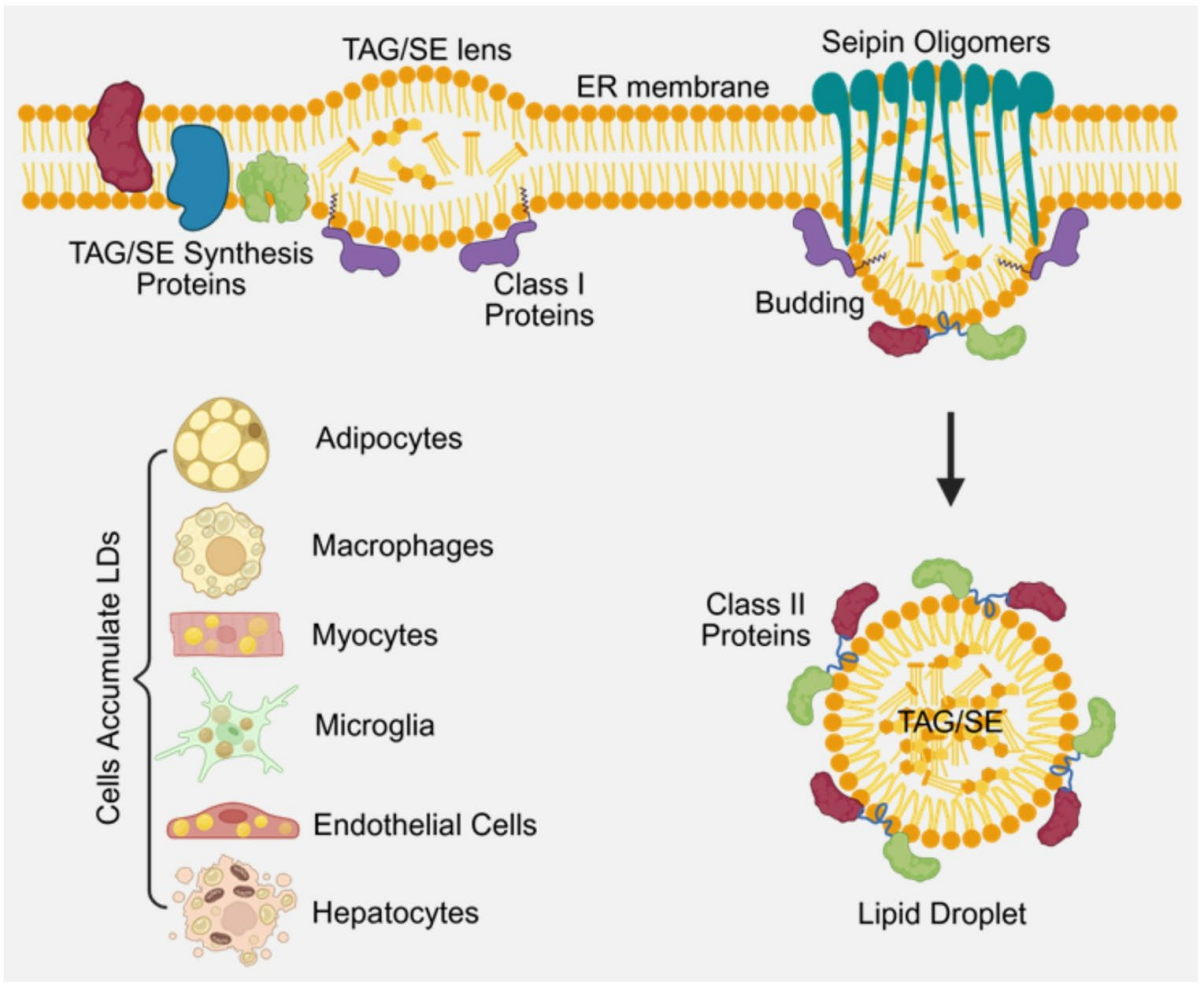
LD biogenesis and distribution in different cell types. TAG and SE are synthesized in the ER’s intramembranous space through a series of biochemical reactions. With demixing and coalesce, a lens-like structure forms and grows, which creates tension in the inner ER membrane. Several ER-resident proteins facilitate the enlargement of the dense structure. When their sizes reach a certain threshold, the anchoring Seipin oligomer protein guides the recruitment of additional proteins to the newly formed LDs. Eventually, these LDs detach from the outer membrane of the ER into the cytosol and move to other subcellular sites through the microtubular networks

### Lipid droplet biogenesis

As shown in Fig. 1, in the ER’s intramembranous space, TAG and SE are synthesized through a series of biochemical reactions (the Kennedy pathway) in a stepwise fashion with participation of several important enzymes including long-chain-fatty-acid-CoA ligase 3 (ACSL3), glycerol-3-phosphate acyltransferase 4 (GPAT4), diacylglycerol acyltransferase 1,2 (DGAT1,2), ancient ubiquitous protein 1 (AUP1), and UBX domain-containing protein 8 (UBXD8) [7]. With demixing and coalesce of TAG, a lens-like structure forms and grows according to the Ostwald ripening mechanism, which creates a tension in the inner ER membrane in the vicinity of the lens structure [8]. Several ER-resident proteins, including the fat storage-inducing transmembrane protein (FIT), perilipin family proteins (PLINs), and the anchoring Seipin oligomer protein [9], facilitate the enlargement of these TAG and SE dense structures. When their sizes reach a certain threshold, the anchoring Seipin oligomer protein guides the recruitment of additional proteins to the newly formed LDs. In this bulging process, different classes of LD-specific proteins are recruited to the monolayer phospholipid membrane, e.g. class I and class II LD proteins. The class I proteins are ER-residential or association proteins petitioning on the bridges between ER and LDs with their hydrophobic hairpins inserted into the bilayer phospholipid membrane of the ER and monolayer phospholipid membrane of LDs [10]. The class II proteins, such as PLINs, comparative gene identification-58 (CGI-58), and adipose triglyceride lipase (ATGL), are directly recruited from the cytosol and bind to the monolayer phospholipid membrane of LDs mainly through their amphipathic α-helices on the portion of the membrane where there are defects in phospholipid packing [11]. Other interacting mechanisms between the class II proteins and the monolayer phospholipid membrane of LDs have also been proposed, such as interaction with LD coating proteins, lipid anchors, and even the core lipids [12]. Eventually, these LDs detach from the outer membrane of the ER into the cytosol and move to other subcellular sites through the microtubular networks.

### Lipid droplet growth

From budding off from the ER to entering the cytosol, LDs go through continuous growth and enlargement through three identified mechanisms. The first is the continuous influx of TAG or SE through the membrane bridges between the ER and LDs, which is mainly facilitated through class I proteins [13]. The second is the continuous synthesis of TAG through DGAT1 proteins located on the surface of the monolayer phospholipid membrane of LDs. The third is through LD fusion which is governed by designated signaling cascades through the Rab GTPases and facilitated by PLINs [14]. While these newly formed LDs also seem to go through a fission process to reduce their sizes, the underlying mechanisms are unknown. Furthermore, why only a selected population of the LDs detach from the ER is also unclear.

Interestingly, LD fusion occurs when the cell death-inducing DFFA-like effector family proteins (CIDEA, CIDEB, and CIDEC) on the monolayer phospholipid membrane form a contact bridge when two LDs meet at the LD contact sites (LDCS) and form a micro-tunnel for the transfer of TAG or SE from small LDs to large ones due to the differences of surface tension [15]. The class II protein PLINs are found in these LDCS sites to facilitate pore formation. As a regulator of CIDE-formed lipid transition pore, the GDP-bound small GTPase Rab8a activates while the GTP-bound Rab8a inhibits LD fusion of small LDs to large ones [16]. The mediation of the equilibrium between GTP-bound Rab8a and GDP-bound Rab8a is controlled by the guanine nucleotide exchange factor (GEF) and GTPase-activating protein (GAP) [17]. In addition, the GDP dissociation inhibitor (GDI) also plays an important role in keeping GDP bound to Rab8a and therefore favors the fusion of LDs [18]. The fusion always occurs directionally from smaller LDs to larger ones as the surface tension of the smaller ones is larger due to the high surface-to-volume ratio and the higher internal pressure of the smaller LDs determined by the Laplace equation [19]. When fusion is completed, the destination of the proteins and phospholipids on the smaller LDs are unknown, as they cannot be simply transferred to the large ones since the surface-to-volume ratio of the large ones is smaller, and as a result, these proteins and phospholipids would be crowded out and dissipated into the cytosol [20].

### Lipid droplet lipolysis

Intracellular lipids are sequestered and stored in LDs when in surplus and are hydrolyzed to FFAs to provide cellular energy when metabolic needs arise. The monolayer phospholipid membrane of LDs is decorated with proteins that have specific functions in the dynamics of lipid turnover. The recruitment and detachment of these proteins to and from the membrane follow the macromolecular crowding mechanism, e.g. when the surface of a LD increases, proteins are recruited, and when the surface decreases, proteins are shed off or crowded out [21]. These proteins and enzymes take cues from cellular metabolic status and determine the routes of lipid dynamics, either lipolysis or sequestration and storage. During nutrient deprivation, the FFAs generated in this process support the cellular energy needs in metabolic tissues. During nutrient surplus, extra FFAs are stored in neutral LDs to prevent lipotoxicity to various subcellular organelles. During cell growth, the phospholipids on the membrane may also support the need for cellular membrane expansion [22].

**Fig. 2:**
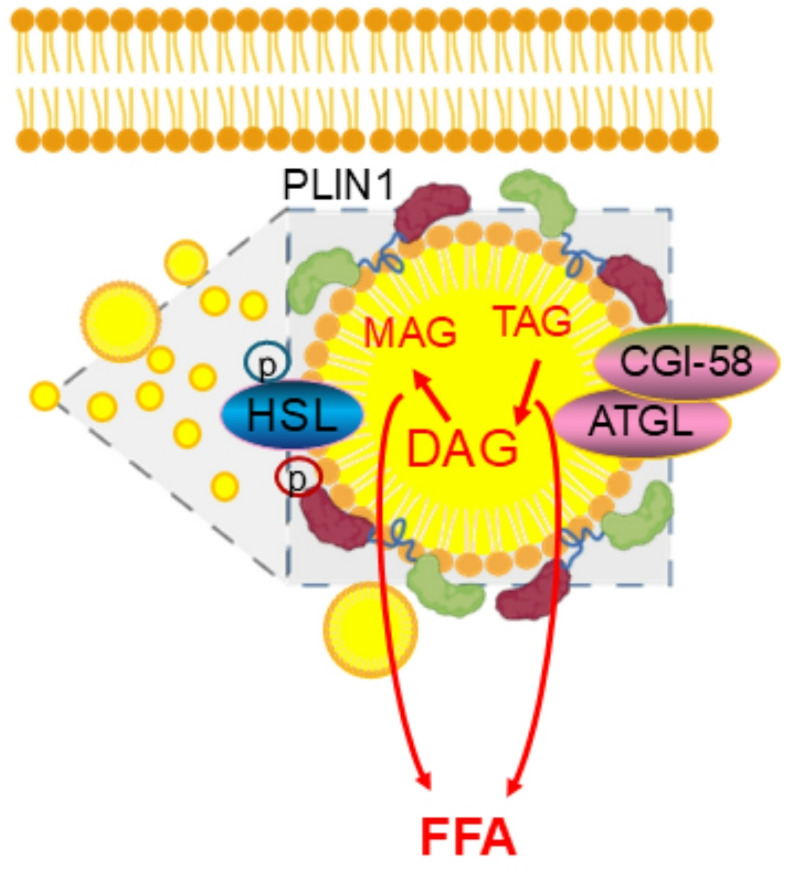
Basal and stimulated lipolysis in LDs. Activated by CGI-58, ATGL hydrolyzes TAG to DAG releasing one FFA. Phosphorylated by protein kinases such PKA, p-HSL and p-PLINs hydrolyze DAG to MAG releasing another FFA. Finally, MAGL hydrolyzes MAG releasing the last FFA

Classical LD lipolysis is a complex process with multiple membranal, and cytosolic proteins as shown in Fig. 2. The PLINs are recognized as coating proteins to protect the LDs from degradational lipolysis and play an important role in the biogenesis and turnover of the LDs. In adipocytes, PLIN1 is the essential LD coating protein which shields the surface of LDs and prevents the basal lipolysis by restricting the access of ATGL to TAG inside the LDs [23]. Any alterations in protein abundance of PLIN1 or its phosphorylation status would result in a change in the dynamics of lipolysis as reported in our previous study [3]. Under basal conditions, ATGL, activated by CGI-58 which interrupts the contact of PLINs to ATGL, hydrolyzes TAG to DAG and releases one FFA, the so-called basal lipolysis. Activated by various hormones, protein kinases such as cAMP-dependent protein kinase A (PKA) phosphorylate hormone-sensitive lipase (HSL), which hydrolyzes DAG to monoacylglycerol (MAG) and releases another FFA in a process known as stimulated lipolysis. The phosphorylation of PLINs (p-PLINs) could also displace its association with CGI-58, therefore, inhibits basal lipolysis but enhances stimulated lipolysis [23]. Furthermore, p-PLINs are required for stimulated lipolysis, i.e. phosphorylation of HSL is dependent on p-PLIN1 for activity [24]. In the last step of LD lipolysis, monoacylglycerol lipase (MAGL) hydrolyzes MAG to glycerol and releases another FFA to complete the whole lipolysis process.

### Lipid droplet lipophagy

**Fig. 3:**
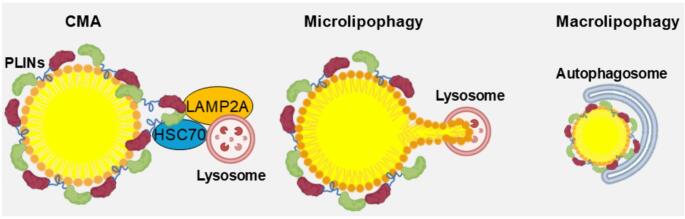
Degradation of the coating proteins and lipids in LDs through CMA, microlipophagy, and macrolipophagy. In CMA, HSC70 recognizes LD coating proteins and binds to the receptor LAMP2A on the surface of lysosome. Subsequently, these proteins are transported to the lumen of lysosome and degraded by LAL. In microlipophagy, lysosome directly pinches off a small portion of LD and degrades it. In macrolipophagy, small LDs are engulfed by autophagosome and subsequently merged with lysosome for degradation

Besides classical lipolysis, lipophagy is another important mechanism to degrade the stored lipids in LDs and to release FFAs for cellular energy homeostasis [25]. Lipophagy is defined as autophagy with lipid products as the content of the phagosomes. There are three types of autophagic mechanisms to degrade proteins coating the monolayer phospholipid membrane and the lipids stored in the LDs: chaperone-mediated autophagy (CMA), microlipophagy, and macrolipophagy as shown in Fig. 3. The CMA is responsible for the degradation of coating proteins such as PLINs, CGI-58, and ATGL [26]. These proteins are recognized by CMA through heat shock cognate 70 kDa protein (HSC70), which is delivered to the lysosomes and ligated to the surface receptor lysosome associated membrane protein 2 A (LAMP2A) and transported into the lumen of lysosomes [27]. Following the degradation of coating proteins such as PLINs by CMA, ATGL and hormone sensitive lipase (HSL) could readily access the content of LDs. Therefore, CMA functions upstream of microlipophagy and macrolipophagy and contributes to LD lipolysis. Subsequently, microlipophagy and macrolipophagy are enabled in the lipid degradation of LDs [28]. In microlipophagy, the lysosomes contact LDs and directly pinch off a portion of the LDs and induce lipid transfer to the lumen of lysosomes where lysosomal acid lipase (LAL) degrades them to FFAs [29]. In macrolipophagy, the autophagosomes engulf small-sized LDs and merge them with lysosomes for the degradation of lipids and associated proteins to replenish protein and lipid sources for cellular function and energy production. The coordination and relative contribution of each of these pathways varies depending on cell type, nutrient status, and disease state [30]. Nonetheless, other proteins such as p38 MAP kinase interacting protein (P38IP) and Atg9 are also implicated in macrolipophagy [3]. These lipophagic machineries, through mechanistically different forms of lipolysis, work together to degrade lipids in LDs and shuttle the lipid products to the sites that are metabolically required for cellular energy and protein homeostasis.

### Lipid droplet transportation in the cytosol

The LDs detached from the outer ER membrane are coated with a repertoire of proteins on the monolayer phospholipid membrane. These proteins and some of the LD membranal phospholipids, such as phosphatidic acid (PA) and phosphatidylinositol 4-phosphate (PI4P), coordinate the bidirectional transportation of LDs on the microtubular network to and from various subcellular locations, where they provide essential FFAs as an energy source during nutrient deprivation or sequester extra lipids during nutrient and energy surplus [31]. The contact partners of LDs such as lysosomes, peroxisomes, and mitochondria as well as the protein composition of the contact sites with LDs have been extensively studied with unique regulatory signaling pathways involved. It has been demonstrated that in cardiomyocytes, the interactions between LDs and microtubules are governed by several proteins and a long non-coding RNA (IncRNA) [32]. The bidirectional movements of LDs are mediated by protein kinases such as AMPK depending on cellular energy status. LDs transported to the mitochondria bind to the receptor PLIN5 on the outer mitochondrial membrane in cardiomyocytes and provide lipids as an essential source to meet the energy requirement [31].

## Mechanistic roles of lipid droplets in major metabolic diseases

### Obesity and insulin resistance

LDs are dynamic cytoplasmic organelles present in almost all cell types as a storage of neutral lipids. Besides its versatile roles in many biological processes, LD hypertrophy and hyperplasia are known key pathological features of obesity. The TAGs and SEs are stored in unilocular LDs in adipocytes sustaining whole-body energy requirement from breakdown of TAGs to DAGs and MAGs to glycerol with FFAs as the fuel [33]. Obesity is associated with increased LD accumulation, dysregulated lipolysis, and chronic inflammation [3]. The low-grade chronic inflammation leads to pro-inflammatory conditions and metabolic diseases, such as insulin resistance, type II diabetes, atherosclerosis, and cardiovascular disorders [34]. In the progression of this chronic inflammatory condition, macrophages are the predominant cell type infiltrated into target tissues to release pro-inflammatory cytokines. These macrophages are known to accumulate excessive multilocular LDs, mainly due to imbalances between lipid synthesis and catabolism. Lipid hydrolyzation is mediated through both the canonical lipolytic pathway and lipophagy. Studies have shown that high fat diet (HFD)-induced obesity is linked with DAG accumulation in cells such as adipocytes, macrophages, and hepatocytes. It has been demonstrated that accumulation of DAG and activation of the noncanonical PKC inflammatory signaling inhibits insulin signaling and induces insulin resistance and diabetes [35]. It is postulated that the dysregulated lipolysis, manifested as increased basal lipolysis and decreased stimulated lipolysis, would increase DAG and activate both PKCδ and caspase recruitment domain containing protein 9 (CARD9). Activation of CARD9 would phosphorylate p38 MAPK that binds to p38-interacting protein (p38IP) and inhibit p38IP-Atg9-facilitated macrolipophagy [16]. The impaired macrolipophagy would further increase the numbers of small LDs and ensure a vicious cycle, propelling the underlying inflammation and exacerbating disease progression. Therefore, dysregulated lipolysis with excessive DAG build-up may be a key mechanism in the development of chronic inflammation in obesity and associated metabolic diseases [3].

### Myocardial and skeletal muscle metabolic disorders

The role of LDs in fatty acid metabolism and energy homeostasis in skeletal muscle and cardiomyocytes has been extensively investigated [36]. Under hyperlipidemic conditions, excessive LDs accumulate in myocytes which exert a detrimental effect on the metabolic equilibrium in these cells [37]. It has been demonstrated that LDs are in contact with mitochondria through PLIN5, a specific perilipin family protein in cardiomyocytes [38] and skeletal muscle cells [39]. PLIN5 serves as a receptor of mitochondria to LDs and brings additional proteins, such as Rab8a GTPases, to the contact sites to regulate energy production under the control of energy status-sensing protein kinases, such as 5’-monophosphate-activated protein kinase (AMPK) [40]. The GTP-bound Rab8a is the active form of the GTPase that regulates the contact of LDs with mitochondria in the myocytes while the GDP-bound Rab8a is the active form which regulates the interaction and fusion of LDs [40]. This signaling may provide a bidirectional control mechanism in the energy homeostasis of metabolically active myocytes. When nutrients are deprived of cardiomyocytes or during exercise in skeletal muscle cells, AMPK is activated. The activated AMPK converts the GDP-bound Rab8a to the GTP-bound GTPase and facilitates the contact of LDs to mitochondria to provide FFA for ATP production through acylcarnitine on the outer membrane of mitochondria [32]. When nutrient deprivation is prolonged, the mitophagy machinery is activated to degrade the stressed mitochondria and other cellular organelles to replenish the sources of FFA and proteins [41]. Recent studies have demonstrated that during this prolonged energy scarcity, LDs can envelop the mitophagy-targeted mitochondria and play a protective role by sustaining FFA release for cell survival [42]. Conversely, when nutrients are in surplus, AMPK is deactivated. Then the shift from the GTP-bound Rab8a to the GDP-bound GTPase would detach the LDs from mitochondria and fuse them with other LDs to sequester the surplus FFA into storage.

In addition, the activation status of AMPK also determines the dispersion and refocusing of LDs through the microtubular network to and from the contact sites of mitochondria [31]. In this unique transportation process, lipid droplet-associated IncRNA lipid transporter (LIPTER) and PI4P have been recognized as tethering proteins to facilitate the binding and moving of LDs along the guided microtubular networks. While the LD dynamics may directly contribute to the metabolic disorders in these cells, the dysregulated LD lipolysis in other cells and tissues also contribute to the overall inflammation status in a paracrine manner leading to inflammatory remodeling and adaptive response and metabolic disorders [3].

### Endothelial and vascular abnormalities

Endothelial cells and other vascular cells play critical roles in regulating vascular tone and normality in the vasculature correlating with metabolic diseases such as atherosclerosis and ischemic injuries [43]. The role of LDs in endothelial cells is demonstrated through several recent studies under various vascular inflammatory conditions [44]. Due to their unique inflammatory environment, these vascular LDs contain highly unsaturated lipids [45]. Therefore, lipolysis or lipophagy of these LDs would release the unsaturated lipid species, resulting in production of inflammatory markers such as prostaglandin I2 (PGI_2_) [46]. While the mechanisms for the accumulation and distribution of LDs in endothelial cells are not fully understood, their biogenesis processes are suggested to follow similar pathways as in other cell types [47]. These studies further demonstrated that the accumulation of LDs may destabilize eNOS mRNA therefore reducing NO production and adversely regulating vascular tone with serious implications for vasoconstriction and hypertension [44]. In addition, the accumulation of LDs has been implicated in the activation of inflammatory transcription factors such as the nuclear factor kappa B (NF-κB) and its down-stream genes, making the endothelium lining more attractive to leukocytes and prone to atherogenesis^46^. These cascade events eventually lead to plaque formation and rupture, causing blockade of blood and nutrients and ischemic tissue damage. However, the specific signaling pathways underlying the accumulation of LDs to the activation of inflammatory transcription factors are still not completely understood. Future studies are warranted.

In addition to accumulation in endothelial cells, LDs are also abundantly present in vascular foam cells, where the main content of the lipids are cholesterols and esterified cholesterols [48]. Systemic pressure alterations as well as local triggers can induce LD accumulation and acceleration of foam cell formation in smooth muscle cells [49]. Therefore, lipolysis or lipophagy of the lipids in these LDs should be anti-atherogenic in protecting the vascular wall from the development of atherosclerosis and vascular dysfunction.

It is worth noticing that in the context of excessive lipid accumulation, other lipid products such as ceramides [50], oxidized phospholipids [51], eicosanoids [52], and lipid peroxidation products [53] may still play major roles to incur lipotoxicity and cause endothelial and vascular dysfunction.

### Non-Alcoholic fatty liver disease

The liver is the major organ in handling and transporting lipids to the whole body for metabolic requirements. LDs are major lipid storage and buffering organelles in the hepatocytes which play a critical role in lipid homeostasis in the liver and throughout the whole body. The accumulation of LDs is the hallmark of non-alcoholic fatty liver disease and steatosis accompanied by inflammation, fibrosis, and cirrhosis [54]. Therefore, understanding of the LD dynamics has important implications for therapeutic interventions for related liver lipid storage diseases. The dynamics of LD growth, fusion, and degradation are controlled by intricate signaling pathways. Under normal nutrient conditions, the CIDEB subtype family of proteins form micro tunnels between two adjacent LDs, which is facilitated by PLINs in the hepatocytes [55]. However, under well-fed nutrient surplus conditions, all forms of CIDEs (CIDEA, CIDEB, and CIDEC) are involved in the growth of LDs and expansion of LD repertoire [56]. It is the GDP-bound Rab8a GTPase that activates CIDEs and opens the micro tunnels and transfers FFA from smaller LDs to larger ones driven by the difference in surface tension [15]. In the hepatocytes, the Akt substrate 160 (AS160) serves as a GAP to dephosphorylate the GTP-bound Rab8a to the GDP-bound Rab8a, while the mammalian suppressor of Sect. 4 (MSS4) serves as a GEF to reverse this conversion [14]. Uncontrolled LD hypertrophy and hyperplasia eventually lead to non-alcoholic fatty liver disease and steatosis which compromises liver function.

### Neurodegenerative disorders

It has long been recognized that multi-lobular LDs accumulate in microglia (LDAM) in aging patients with neurogenerative disorders [57]. Only recently have the accumulated LDs in microglia and other brain cells been recognized for contributing to the impairment of neuronal dysfunction and inflammation underlying Alzheimer’s disease (AD), Parkinson’s disease (PD), Huntington’s disease, amyotrophic lateral sclerosis (ALS), multiple sclerosis (MS), and other neurodegenerative disorders [4–6,58,59]. As a key regulator of lipid transport and metabolism, ApoE4/4 has been linked to the pathological genesis of LDs [60]. Taking AD as an example, the activated microglia underscore the detrimental effects of neural inflammation in the disease progression [58]. In the late stages of AD, the hypertrophied and hyperplastic LDs in the microglia inadvertently incur dysregulated lipolysis in small LDs due to their increased surface area over volume [3]. Therefore, studies on the mechanistic roles of LD lipolysis or lipophagy should enhance our current understanding of the disease pathology. We speculate that the dysregulated lipolysis, manifested as increased basal lipolysis and decreased stimulated lipolysis, may increase neuroinflammation. With excessive lipid accumulation, other lipids products such as ceramides, oxidized phospholipids, eicosanoids, and lipid peroxidation products may also incur lipotoxicity and exacerbate disease progression [50,51]. Therefore, suppressing dysregulated lipolysis and restoring lipophagy would deplete the small LDs responsible for neuroinflammation. Further studies are warranted for a systematic understanding of these postulated functions of LDs in the brain.

### Potential therapeutic targets

As our understanding of the mechanistic roles of LDs in various disease conditions increases, the potential for therapeutic interventions based on these mechanisms has also emerged. In basal lipolysis, the key lipase ATGL hydrolyzes TAG to DAG and FFA. Therefore, to correct the dysregulated lipolysis, drugs targeting ATGL should prove to be therapeutically useful to curb the underlying inflammation associated with excessive LD accumulation. In stimulated lipolysis, several protein kinases play critical roles in phosphorylating HSL and PLINs to facilitate the conversion of DAG to MAG and FFA. Therefore, agonists of these protein kinases should alleviate the buildup of toxic lipid byproducts and further support cellular function and survival when energy sources are scarce. To test if DAG accumulation drives sustained inflammation and downstream pathological effects, we have employed Atglistatin (Atg), an ATGL inhibitor, to successfully deplete the accumulation of DAG and suppress its downstream inflammatory responses. With the understanding of the dynamics of LD fusion, one can envision potential targets to inhibit or promote the fusion process depending on the cellular energy and redox status. As AMPK facilitates the production of FFA from LDs in contact with mitochondria, its agonists should support energy production and cellular function. Recently, it has been demonstrated that CARD9 plays a critical role in regulating the dysregulated lipolysis in macrophages in obesity and associated cardiovascular dysfunction. Therefore, potential small molecule CARD9 inhibitors should also prove to be important in treating obesity-associated chronic inflammation and cardiovascular abnormalities originating from LD accumulation.

## Conclusion remarks

In summary, as an emerging research field LDs have garnered a renewed interest in their cellular functions in various cells and tissues. From the concept as an inert lipid storage unit to recent discoveries of their dynamic functionality, our understanding of their roles in various disease conditions will continue to bring in new technologies and mechanisms into this very important research area. LDs have also emerged as an important player in cellular lipid and protein handling that may not have a direct relationship with the above discussed mechanisms and conditions. There are still many unanswered questions regarding the fission of LDs, the specific lipid compositions and related interactions in the LDs dynamics, and the consequences of LDs accumulation on cellular morphology. Some interesting hypotheses have been proposed as to if LD fusion may be able to serve as a mechanism to repair the damaged cell membrane as the monolayer phospholipid membranes of LDs and the bilayer phospholipid membranes of cells have the same origin from the ER. Further studies are needed to capitalize on the uncovered mechanisms to develop practical therapeutic interventions to combat human disease where lipids are always present either in excess or deprivation.

## Data Availability

No datasets were generated or analysed during the current study.

## Abbreviations

LD: Lipid droplet
TAG: Triglyceride
DAG: Diacylglycerol
MAG: Monoacylglycerol
ACSL3: Long-chain-fatty-acid-CoA ligase 3
GPAT4: Glycerol-3-phosphate acyltransferase 4
DGAT1,2: Diacylglycerol acyltransferase 1,2
AUP1: Ancient ubiquitous protein 1
UBXD8: UBX domain-containing protein 8
FIT: Fat storage-inducing transmembrane protein
PLINs: Perilipin family proteins
CGI-58: Comparative gene identification-58
ATGL: Adipose triglyceride lipase
CIDEA, CIDEB, and CIDEC: Cell death-inducing DFFA-like effector family proteins A, B, and C
LDCS: LD contact sites
GEF: Guanine nucleotide exchange factor
GAP: GTPase-activating protein
GDI: GDP dissociation inhibitor
HSL: Hormone-sensitive lipase
MAGL: Monoacylglycerol lipase
CMA: Chaperone-mediated autophagy
HSC70: Heat shock cognate 70 kDa protein
LAMP2A: Lysosome associated membrane protein 2 A
LAL: Lysosomal acid lipase
P38IP: p38 MAP kinase interacting protein
PI4P: Phosphatidylinositol 4-phosphate
IncRNA: Long non-coding RNA
LIPTER: Lipid droplet-associated IncRNA lipid transporter
MSS4: Mammalian suppressor of Sect. 4
LDAM: LDs accumulate in microglia
CARD9: Caspase recruitment domain containing protein 9
